# The Relationship Between Physical Housing Characteristics, Housing Accessibility and Different Aspects of Health Among Community-Dwelling Older People: A Systematic Review

**DOI:** 10.1177/08982643231175367

**Published:** 2023-05-18

**Authors:** Christina Heller, Maria Haak, Steven M. Schmidt, Carlos Chiatti, Lisa Ekstam, Maria H. Nilsson, Björn Slaug

**Affiliations:** 1 Department of Health Sciences, 5193 Lund University, Lund, Sweden; 2 Department of Nursing Education and Integrated Health Sciences, 4342 Kristianstad University, Kristianstad, Sweden; 3 R&D Unit, Tech4Care srl, Falconara Marittima, Italy; 4 Clinical Memory Research Unit, Department of Clinical Sciences Malmö, 5193 Lund University, Lund, Sweden; 5 Memory Clinic, Skåne University Hospital, Malmö, Sweden

**Keywords:** physical housing characteristics, housing accessibility, ageing population, health

## Abstract

**Objectives:** To synthesize the evidence on the relationships between physical housing characteristics or housing accessibility and different aspects of health among community-dwelling people 60 years and older. **Methods:** A systematic review of recent evidence with a narrative synthesis was conducted. **Results:** We included 15 studies and found three themes covering physical housing characteristics or housing accessibility that are associated with aspects of health among community-dwelling older adults: (1) interventions by home modifications targeting housing features both at entrances and indoors; (2) non-interventions targeting indoor features; (3) non-interventions targeting entrance features, that is, the presence of an elevator or stairs at the entrance. The overall quality of evidence across studies was assessed as very low. **Discussion:** The findings highlight the need for studies with a stronger research design and higher methodological quality that address the physical housing environment in relation to health among older adults to strengthen the body of evidence.

## Background

Though housing issues for the aging population (aged 60 years and older) have been recognized as an important Public Health challenge for a long time, the COVID-19 pandemic contributed even further to expose the housing situation of older people. As the pandemic forced people to stay in their homes for most of the time, the often poor and inadequate housing conditions many older people live in were made more visible and apparent (Delclós & Vidal, 2021; Housing Europe, 2021). This underlined the urgent need to defend and reinforce the right to adequate and affordable housing, especially for the aging population. Notwithstanding the pandemic, individuals in developed economies tend to spend more than 90% of their time indoors (Palacios et al., 2020). The conditions and maintenance of the physical housing environment have been associated with several aspects of the population’s health, including frequency of use of medical visits (Palacios et al., 2020), mortality (Damiens, 2020), falls (Lee, 2021), participation (Levasseur et al., 2020; Thordardottir et al., 2020), subjective physical and mental health (Garin et al., 2014) and mental well-being (Guo et al., 2021).

The relationship between physical housing characteristics and health has also been highlighted by the United Nations (UN) Sustainable Development Goals (SDG3 and SDG11) (United Nations, 2021b). Specifically, housing accessibility is mentioned as one of the key characteristics within the SDGs, encompassing the fit between the person´s functional capacity and the demands of the housing environment (Iwarsson & Ståhl, 2003). However, housing accessibility seems, in many countries, to be inadequate for the aging population (Granbom et al., 2016). Even in countries with relatively high housing standards, environmental barriers (e.g., shelves placed extremely high or high thresholds at entrances) are common in the existing housing stock (Iwarsson et al., 2006). This creates difficulties for older adults and people with functional limitations to independently perform activities of daily living (ADL). In order to age in place with maintained independence, a housing environment that is amenable to changes in a person’s functional capacity as one becomes older is required (Lawton, 1986). Because of a higher life expectancy, the world’s population is expected to increase by two billion people, from 7.7 billion at present to 9.7 billion in 2050. Consequently, older persons will outnumber adolescents and youth (ages 15 to 24) by the year 2050 (United Nations, 2021a). As multimorbidity, functional decline, and dependence on mobility devices tend to increase in older age (WHO, 2015a), this demographic shift will have a tangible impact on societal housing planning. However, it is also important to point out, that older people are a heterogeneous group with various levels of functional capacity and different needs regarding the design of the physical housing environment (Heller et al., 2022; Luther et al., 2019).

Several body functions are affected by the biological aging process, which has consequences for housing needs. For instance, the muscle mass tends to decline with increasing age, which can be associated with reduced strength (Ireland et al., 2021). This age-related decline can affect older people’s well-being, life satisfaction, and quality of life if the housing environment is not designed or adapted to compensate for reduced muscle strength (Boström et al., 2018; Crist, 2000). Loss of vision and hearing are other examples of age-related decline (Evans et al., 2002). A poor fit between functional capacity and environmental demands can in turn lead to dependence in ADL (Iwarsson et al., 2007; Iwarsson & Ståhl, 2003). ADL denotes both personal (i.e., P-ADL, such as transferring, bathing, and eating) and instrumental activities of daily living (i.e., I-ADL, such as managing finances, housecleaning, and managing transport).

In sum, there is plenty of evidence in the literature for a link between the housing environment and health-related outcomes, and this matter has been widely studied. Some reviews of the evidence also exist. Ige et al. (2019) aimed to systematically review research on the impact of buildings on health in the general population. They found that housing refurbishment and modifications, provision of adequate heating, and improvements to ventilation and water supply were associated with improved respiratory outcomes, quality of life, and mental health. Another review from 2014 aimed to summarize the evidence on the built environment (including housing) and health outcomes in community-dwelling people aged 50 years or older, focusing on the three domains of physical health, mental health, and life satisfaction (Garin et al., 2014). A high degree of variability in methodology and results in all three health domains was observed. However, a link between home size, housing type, usability, interior environment, and the health outcomes quality of life, life satisfaction, and well-being was found. Still, a systematic review of the more recent evidence targeting the physical housing environment and health of community-dwelling people aged 60 years and older is lacking.

The WHO has repeatedly highlighted the importance of the health outcomes body functions, well-being, quality of life, activities of daily living, life satisfaction, and social participation as a Public Health priority (WHO, 2001; WHO, 2015a; WHO, 2018). Self-perceived health is widely used as an integrative measure of the biological, mental, and functional dimensions of health (Ocampo, 2010). The objective of this systematic review was to synthesize the more recent evidence on the relationship between physical housing characteristics or housing accessibility and different aspects of health among community-dwelling people 60 years and older. The review focuses on the following aspects of health:

1) body functions: physiological and psychological functions of body systems (WHO, 2001)
2) self-perceived health: subjectively reported overall health (Ocampo, 2010)
3) well-being: subjective experiences of life with respect to satisfaction, pleasant and unpleasant feelings (Diener & Suh, 1997)
4) quality of life: perceived position in life in a cultural and value systems context and in relation to goals, expectations, standards and concerns (WHO, 2012)
5) life satisfaction: subjective positive and negative assessments of life as a whole (Delhey, 2004)
6) ADL: fundamental skills required to independently care for oneself (Katz, 1983)
7) social participation: involvement in activities that provide interaction with others in society or the community (Levasseur et al., 2020).

## Methods

This systematic review was performed according to the recommendations of the “Preferred Reporting Items for Systematic Reviews and Meta-Analyses” 2020 (PRISMA) (Page et al., 2021). The study was registered in the “International Prospective Register of Systematic Reviews” (PROSPERO) in 2022 (CRD42022304471).

### Data Sources and Searches

To capture aspects of health as well as the housing and built environment, a systematic search was conducted in January 2022 using PubMed, Cumulative Index to Nursing and Allied Health Literature (Cinahl), Inspec, and Cochrane Database of Systematic Reviews. The first and last author, together with a librarian, performed a comprehensive search of studies published between 2010 and 2021 to capture more recent studies. The keywords for the search included a combination of terms related to health as well as the built environment, which was altered for each database. We initially identified many studies focusing on Covid-19 vaccines, nutrition, palliative care, assisted living, and oral health, which were not in our research interest. We decided to exclude them by including “NOT” as a search term. The search was limited to the English language and human study participants aged 60 years or older. The age criterion of 60 years was chosen as it is the definition of older people applied by the WHO (WHO, 2015b). For search strategies, see Appendix 1*.*

### Study Eligibility Criteria and Selection

We included randomized controlled trials (RCTs), meta-analyses, literature reviews, quasi-experimental studies, and longitudinal studies. Systematic reviews and meta-analyses were included to screen their reference lists for additional articles. The studies met the inclusion criteria if they (a) focused on the physical housing environment, (b) addressed community-dwelling (c) older adults, aged 60 years and older, and included at least one of the following health outcomes: (1) body functions, (2) self-perceived health, (3) well-being, (4) quality of life, (5) life satisfaction, (6) ADL (P-ADL and/or I-ADL), or (7) social participation. Studies were excluded if they focused on people younger than 60 years, if the intervention was not related to any assessment of the housing environment, and if the study was conducted in long-term care facilities, hospitals, or assisted living. The extracted files were imported into the Rayyan systematic review site for inclusion or exclusion based on the defined criteria (Rayyan, 2022). After removing duplicates, the first and the last author and an external reviewer independently conducted a title and abstract review of the same first 100 studies against eligibility and exclusion criteria. Inconsistencies were discussed to reach agreement on how to practically apply the criteria. After that, the remaining articles were screened individually and categorized into the groups relevant, irrelevant, and maybe. Weekly meetings were held until all discrepancies were resolved. Any discrepancies that arose were resolved by consensus discussions or by consultation with the second co-author when necessary. Articles categorized as irrelevant were excluded. Finally, the first and last author as well as the external reviewer, checked the full texts against inclusion and exclusion criteria.

### Data Extraction

The first author extracted data from the included studies using a customized Microsoft Excel table. Data extraction included type of intervention, study design, method of analysis used, participant characteristics (age, sex, level of education, and sample size), housing characteristics, country in which the study had been conducted, and measures of relationships with the health outcomes (body functions, perceived health, well-being, quality of life, life satisfaction, activities of daily living, and social participation). Additionally, intervention characteristics, such as frequency, duration, and coverage, were extracted. The table with extracted data was then reviewed for completeness and accuracy by the last author. To further validate the data extraction, the table was reviewed and discussed in an iterative procedure with all authors.

### Evaluation of Scientific Evidence

Studies meeting the inclusion criteria were evaluated for quality of scientific evidence in a two-step procedure guided by the GRADE guidelines (Guyatt et al., 2011). In a first step, all authors, except the second author, were involved in joint discussions to rate limitations in the study design for each individual study. If discussions were not conclusive, the second author had the role to resolve any disagreements. Specific criteria were used for study limitations, in accordance with the GRADE guidelines. The following criteria applied for RCTs: lack of allocation concealment, lack of blinding, large loss to follow-up/failure to adhere to an analysis according to the intention to treat principle, stopping a trial early for benefit, and selective reporting of events. For observational studies, the following criteria applied: eligibility, flawed measurement of both exposure and outcome, failure to adequately control confounding, and incomplete or inadequately short follow-up for longitudinal studies. In a second step, the first and the last author rated the quality of evidence for each outcome across studies. In case of disagreements, joint discussions between the first and last author followed until consensus was reached, or if the disagreements were not resolved, the third author, was consulted. The evaluation of scientific evidence was further strengthened by joint discussions involving all authors. It should be noted that by using the GRADE approach, the quality of evidence of observational studies without special strengths (such as large effects or other factors that can increase the quality of evidence) is already categorized as low or very low in comparison to RCTs which are categorized as high or moderate (Guyatt et al., 2011).

### Data Analysis and Synthesis

Data was analyzed using an inductive approach since no established framework to our knowledge included all the health outcomes of our interest. To synthesize and explore the findings in a manner that captures the expected heterogeneity of study designs and methods, a narrative synthesis was used. A narrative synthesis aims to explore and explain relationships within and between studies, that is, differences and similarities in characteristics and findings of the studies (Popay et al., 2006) and was deemed appropriate to make the findings comprehensive and elucidate reasons for the strength or lack of strength of the evidence on the relationships under study. The first author examined the findings and outlined how the data could be synthesized and presented it by regrouping the studies based on a combination of study design and the nature of the exposure, that is how the housing characteristics had been measured or captured. The synthesis was discussed with the second and last author until consensus was reached.

## Results

**Figure 1.**
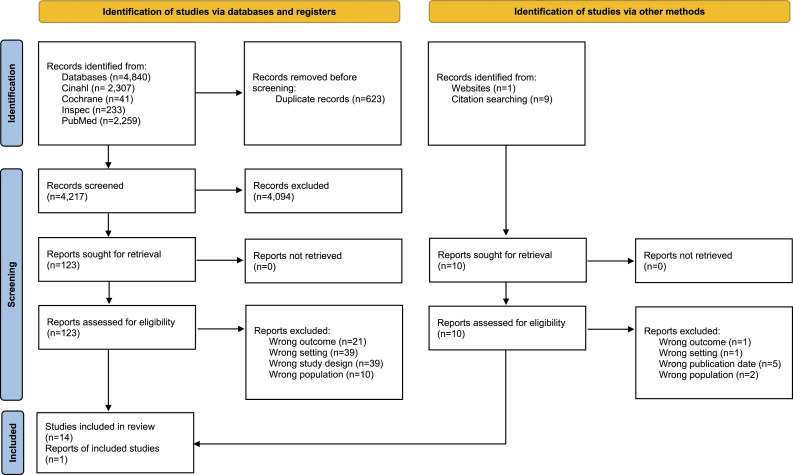
Flow Diagram (Page et al., 2021).

We found 4,840 records from database searches and ten records were identified from citation searches. After duplicate removal, we screened 4,217 records, from which we reviewed 123 full-text documents retrieved from the database search and ten from the website and citation search. Finally, 15 papers were included (Figure 1).

The majority of included articles reported cross-sectional studies (*n* = 9), followed by a few longitudinal studies (*n* = 4) and RCTs (*n* = 2) whereof one was a pilot trial. The included studies captured a diverse range of sample sizes (from *N* = 40 to *N* = 6,578), countries, physical housing characteristics, and health outcomes. See Table 1 for more details regarding study characteristics, and for a summary of the effect sizes, see Appendix 2.

**Table 1.** Basic Characteristics of Reviewed studies Focusing on Housing Characteristics and Health Outcomes (*N* = 15).^a^

No.	First author, publication Year, Country	Study design	Sample: *N*, Age, Women (%)	Exposure (assessment tool)	Health outcomes							
						Body functions *n* = 6	Self-perceived health *n* = 2		Quality of life *n* = 5	Life satisfaction *n* = 2	ADL (P-ADL and I-ADL) *n* = 7	Social participation *n* = 2
1	Clarke, 2014, USA	Cross-sectional study	*N* = 6,578, 65 years and older, Women (56.6%)	Walking surface leading to building; stairs at entrance (study specific)		Difficulty going out (study specific)						
2	García-Esquinas et al., 2017, Spain	Cross-sectional study	*N* = 2,012, 60 years and older, Women (49.6%)	Walk-up building, no elevator (study specific)		SPPB					I-ADL: Lawton & Brody scale	
3	González et al., 2020, Spain	Cross-sectional study	*N* = 79, 60 years and older, Women (78.5%)	Physical home environment, that is, decoration and location of the home (HOME)		Cognitive function (MoCA), Cognitive decline (S-IQCODE)			ICECAP-O			
4	Kim et al., 2021, South Korea	Cross-sectional study	*N* = 2,007, 60 years and older, Women (56.5%)	Perceived housing accessibility and usability (study specific)					To what degree participants were satisfied with their life			
5	Leung et al., 2018, China	Cross-sectional study	*N* = 365, 60 years and older, Women (64.7%)	Indoor built environment, space and distance; building services, for example, lighting, ventilation; supporting facilities, for example, handrails, color; barrier-free design (study specific)				Overall QoL (study specific)			Social relationships (study specific)	
6	Mitoku & Shimanouchi, 2014, Japan	Longitudinal study	*N* = 547, 65 years and older, Women (64.5%)	Housing adaptations, for example, installation of handrails, elimination of floor height differences, change of lavatory basin, change of floor materials, change of door etc. (study specific protocol)						P-ADL: Ability to perform personal ADLs (progression of frailty)		
7	Nakhodaeezadeh et al., 2017, Iran	Cross-sectional study	*N* = 128, 60 years and older, Women (49.2%)	Characteristics of entrance, hall, lounge, kitchen, double bedroom, single bedroom, alternative bathroom, cupboard, general items, and assistive technology (EVOLVE)				CASP-19				
8	Pérez-Hernández et al., 2018, Spain	Longitudinal study	*N* = 2,614, 60 years and older, Not reported	Walk-up building, no elevator (study specific)		SPPB					I-ADL: Lawton & Brody Scale	
9	Slaug et al, 2017, Sweden & Germany	Longitudinal study (with a simulation modeling design)	*N* = 847, aged 80–89 years, Women (75.0%)	Physical barriers in the home environment, for example, high thresholds, narrow door openings, stairs without handrails; measure of housing accessibility problems (Housing Enabler)							I-ADL: Cooking Shopping Cleaning Transport (ADL staircase)	
10	Szanton et al., 2011, USA	Randomized controlled pilot trial	*N* = 40, 65 years and older, Women (95.0%)	Physical barriers in the home environment, for example, holes in floors, uneven carpeting, and lack of railings or banister (Client Clinician Assessment protocol)				EuroQOL EQ-5D			P-ADL: Katz ADL I-ADL: ADL staircase	
11	Taylor et al., 2020, Australia	Randomized controlled trial	*N* = 309, 65 years and older, Women (48.9%)	Home hazards, for example, lack of railings, raised thresholds, lack of lighting (Home Safety Assessment Tool)		PPA SPPB CSPS MBR		EQ-5D-5 L			P-ADL: DAD	
12	Tomioka et al., 2018, Japan	Population-based longitudinal cohort study	*N* = 6,722, 65 years and older, Women (56.5%)	Walk-up building, no elevator (study specific)							I-ADL: TMIG-IC	
13	Tomsone et al., 2013, Sweden, Germany & Latvia	Cross-sectional study	*N* = 1,098, Germany and Sweden: 80–89 years & Latvia: 75–84 years, Not mentioned	Physical barriers in the home environment, for example high thresholds, narrow door openings, stairs without handrails (Housing Enabler)			SF-36 single item					
14	Tsuchiya-Ito et al., 2019, Japan	Cross-sectional study	*N* = 1,928, 65 years and older, Women (60.7%)	Accessibility of housing environment, for example, difficulty entering or leaving the home, unable to climb stairs, difficulty maneuvering within rooms, no railings although needed Healthy Housing Environment			Self-reported health			How would you rate your life satisfaction in the last 3 days?		
15	Yang & Sanford 2011, USA	Cross-sectional study	*N* = 122, 60 years and older, Women (64.0%)	Physical home environment, for example, height and location of toilet (CASPAR)		Mobility limitation, ease-difficulty						PARTS/M

Abbreviations: CASP-19 = Control, Autonomy, Self-Realization and Pleasure scale, CASPAR = Comprehensive Assessment and Solution Process for Aging Residents, CSPS = Continuously scored lower extremity Summary Performance Score, DAD = the Disability Assessment for Dementia, EQ-5D = EuroQol-5 Dimension, ICECAP-O = ICEpop CAPability measure for Older people, I-ADL = Instrumental Activities of Daily Living, MBR = Maximal Balance Range Tests, MoCA = Montreal Cognitive Assessment, P-ADL = Personal Activities of Daily Living, PARTS/M = Participation survey/mobility, PPA = Physiological Profile Assessment, SPPB = Short Physical Performance Battery, S-IQCODE = Spanish Informant Questionnaire on Cognitive Decline in the Elderly, TMIG-IC = Tokyo Metropolitan Institute of Gerontology Index of Competence

^a^Please note: none of the included studies focused explicitly on well-being, therefore, well-being was excluded in Table 1.

### Risk of Bias and Overall Grading of the Scientific Evidence

**Figure 2.**
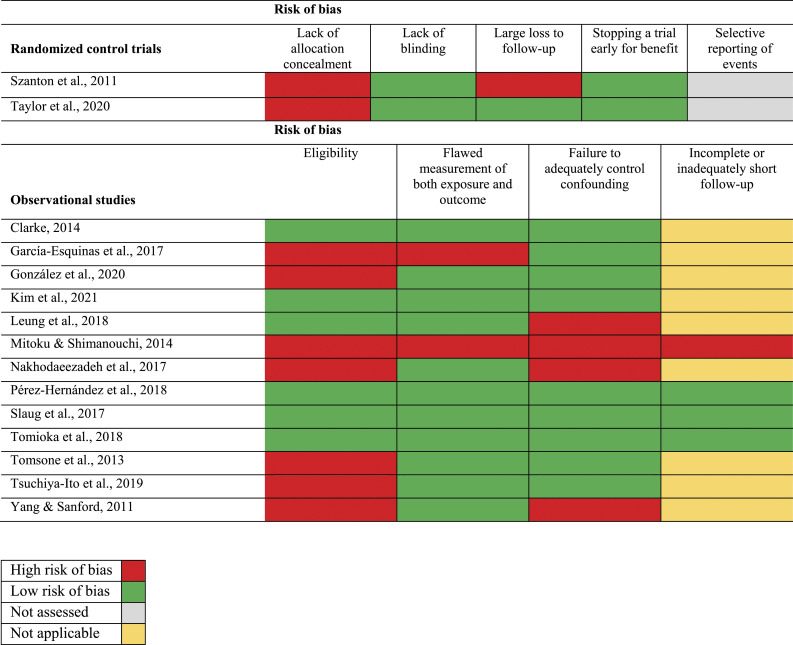
Risk of bias summary.

Only five of the 15 studies showed a low risk of bias for all applicable criteria regarding the study design (Clarke, 2014; Kim et al., 2021; Pérez-Hernández et al., 2018; Slaug et al., 2017; Tomioka et al., 2018). After assessing the quality of evidence for each health outcome (body functions, self-perceived health, well-being, quality of life, life satisfaction, P-ADL and I-ADL, and social participation) across studies, the evidence was assessed as very low for each outcome. This implies that there is very little confidence in the effect estimates (Figure 2, Table 2). No study explicitly addressed well-being; therefore, this health outcome has not been included in the grading of scientific evidence.

**Table 2.** Overall Grading of Scientific Evidence for the Health Outcomes.^a^

		Factors that decrease the quality of the evidence^b^					Factors that increase the quality of the evidence^c^			
Authors	Health outcome	Limitations in study design	Indirectness	Imprecision	Inconsistency	Publication bias	Large effect	Plausible confounders	Dose-response gradient	Overall grading of scientific evidence^d^
Clarke, 2014; García-Esquinas et al., 2017; Pérez-Hernández et al., 2018; Taylor et al., 2020; Yang & Sanford, 2011	Body functions	−1	−1	−1	−1	0	0	0	—	⨁○○○ very low
García-Esquinas et al., 2017; Mitoku & Shimanouchi, 2014; Pérez-Hernández et al., 2018; Slaug et al., 2017; Szanton et al., 2011; Taylor et al., 2020; Tomioka et al., 2018	P-ADL and I-ADL	−1	−1	−1	−1	0	0	0	—	⨁○○○ very low
González et al., 2020; Leung et al., 2018; Nakhodaeezadeh et al., 2017; Szanton et al., 2011; Taylor et al., 2020	Quality of life	−1	−1	−1	−1	0	0	0	—	⨁○○○ very low
Leung et al., 2018; Yang & Sanford, 2011	Social participation	−1	−1	−1	0	0	0	0	—	⨁○○○ very low
Tomsone et al., 2013; Tsuchiya-Ito et al., 2019	Self-perceived health	−1	−1	−1	−1	0	0	0	—	⨁○○○ very low
Kim et al., 2021; Tsuchiya-Ito et al., 2019	Life satisfaction	−1	−1	−1	0	0	0	0	—	⨁○○○ very low

Abbreviations: P-ADL = Personal Activities of Daily Living, I-ADL = Instrumental Activities of Daily Living.

^a^Note: none of the studies focused explicitly on well-being, therefore omitted from this table.

^b^Note: −1 means the quality of evidence was downgraded due to serious concerns by this factor; 0 means the quality of evidence was not downgraded by this factor.

^c^Note: 0 means the quality of evidence was not upgraded by this factor.

^d^Note: The overall quality of evidence is graded from very low to high.

### Synthesis of the Results

By synthesizing study design and exposure, we found that the three intervention studies focused in detail on housing characteristics, while the twelve non-intervention studies focused on indoor or entrance features in a simpler fashion. We, therefore, categorized the studies into three themes: (1) interventions by home modifications targeting housing features both at entrances and indoors; (2) non-interventions targeting indoor features; (3) non-interventions targeting entrance features, that is, the presence of an elevator or stairs at the entrance.

### Interventions by Home Modifications

Three studies addressed interventions by home modifications (Mitoku & Shimanouchi, 2014; Szanton et al., 2011; Taylor et al., 2020) and measured the effects on health outcomes at follow-up. Primary and secondary outcomes were defined in study protocols for two of the studies (Szanton et al., 2011; Taylor et al., 2020). The first study was a cohort study (Mitoku & Shimanouchi, 2014) where health outcomes were compared between participants who got their homes modified (at least one of the modifications installation of handrails, elimination of floor height differences, change of lavatory basin, change of floor materials or change of doors) and those who did not. Follow-ups were conducted after 1, 2, and 3 years. The second study was a pilot RCT (Szanton et al., 2011), and the third was a RCT (Taylor et al., 2020). In the study by Szanton et al. (2011), health outcomes were compared between an intervention group that received modifications to their home based on the study-specific protocol CAPABLE (Occupational therapy protocol of potential physical housing issues), such as holes in the floor, raised thresholds, poor lighting, etc., and a control group that did not. The control group received sessions of attention control. A follow-up was conducted after 24 weeks. In the study by Taylor et al. (2020), health outcomes were compared between the intervention group that received home modifications based on a home hazards checklist (Clemson et al., 1999) and a control group that received care as usual. Follow-ups were conducted after 6 and 12 months.

All three intervention studies focused on P-ADL and one also on I-ADL. The study by Mitoku & Shimanouchi (2014) used the care level (which is based on a professional assessment of the dependence in P-ADL activities) as an indicator of the ability to perform P-ADL. To measure the effect of home modifications, the number of participants that decreased in ability to perform P-ADL (defined as changing to a care level with more P-ADL support) or died, were compared between the intervention and the control group. It was found that fewer people decreased in P-ADL ability in the intervention group at all follow-ups, but the differences were not significant. In the study by Szanton et al. (2011), the intervention group reported fewer difficulties in P-ADL and I-ADL at follow-up, and Cohen’s D effect sizes were estimated to .63 for P-ADL and .62 for I-ADL. Taylor et al. (2020) used regression analysis to estimate between-group differences in P-ADL after 12 months (P-ADL was not assessed at 6-month follow-up), and though a positive tendency to effect home modifications was noted, it was not statistically significant.

Two of the intervention studies compared quality of life between the intervention and control group (Szanton et al., 2011; Taylor et al., 2020). Both studies used the EQ-5D (EuroQol-5 Dimension) as a measure and Szanton et al. (2011) used the EuroQOL as an additional measure. Szanton et al. (2011) reported improvements in EQ-5D and EuroQOL for the intervention group (Cohen’s D .48 and .89, respectively). Taylor et al. (2020) did not find any statistically significant difference between the intervention and the control group with regard to EQ-5D.

Additionally, Taylor et al. (2020) compared body functions between the intervention and control group using four different measures, PPA (Physiological Profile Assessment), SPPB (Short Physical Performance Battery), CSPS (Continuously scored lower extremity Summary Performance Score), and MBR (Maximal Balance Range Tests), but found no statistically significant differences.

### Non-interventions Targeting Indoor Features

In total, eight non-intervention studies addressed indoor features (Gonzáles et al., 2020; Kim et al., 2021; Leung et al., 2018; Nakhodaeezadeh et al., 2017; Slaug et al., 2017; Tomsone et al., 2013; Tsuchiya-Ito et al., 2019; Yang & Sanford., 2011).

Two cross-sectional studies investigated the physical housing environment and body functions (Gonzáles et al., 2020; Yang & Sanford., 2011). Gonzáles et al. (2020) found that homes that were free of barriers, such as stairs with no railings inside the home and slippery floors, were associated with a significantly lower risk of cognitive problems among older adults (OR .95; CI .92 to .96). Yang & Sanford. (2011) found that fewer physical barriers were significantly associated with fewer mobility limitations (OR range from .257 to .627; CI not reported).

Three cross-sectional studies focused on quality of life and indoor physical housing by either using the ICECAP-O (ICEpop CAPability measure for Older people) (Gonzáles et al. (2020)), CASP-19 (Control, Autonomy, Self-Realization, and Pleasure scale) (Nakhodaeezadeh et al., 2017) or a study specific measure targeting the overall QoL (Leung et al., 2018). Gonzáles et al. (2020) found a statistically significant association between a physical housing environment that is free of potential hazards such as stairs with no railings and overcrowded furniture, and a higher quality of life (OR 6.54; CI 1.75 to 24.46). A positive correlation (Corr coeff 0,279)^1^ could also be found between lack of deficiencies in the entrance, hall, kitchen, etc., and higher quality of life of older adults in the study by Nakhodaeezadeh et al. (2017). Leung et al. (2018) found a positive association between accessible furniture and fixtures, lighting, and higher quality of life (Est. Furniture and fixtures .092; Est. Lighting .149). The presence of handrails in the living and sleeping room influenced the overall quality of life of older adults negatively (Est. Handrails −.125).

Two cross-sectional studies investigated life satisfaction with study-specific items on indoor physical housing characteristics. Kim et al. (2021) focused on the degree of participant satisfaction with their life, and Tsuchiya-Ito et al. (2019) focused on how participants rated their life satisfaction in the last three days. A higher level of perceived housing accessibility and usability was significantly associated with a higher level of life satisfaction (Est. .22; CI -.015 to .04)^2^ (Kim et al., 2021). No association could be found between lower life satisfaction and inaccessible housing (e.g., absence of railings, unable to climb stairs) in participants with a high ADL dependence and low ADL dependence (Tsuchiya-Ito et al., 2019).

Furthermore, one longitudinal study focused on I-ADL (Slaug et al., 2017), and demonstrated that older adults living in less accessible housing in Sweden and Germany were more likely to become I-ADL dependent (except for dependence in cleaning in the German sample) in comparison to participants living in accessible housing (OR range from 1.003 to 1.011 (CI 1.000 to 1.015)).

Two cross-sectional studies investigated the indoor housing environment and social participation by either focusing on social relationships (Leung et al., 2018) or community participation (Yang & Sanford, 2011). Leung et al. (2018) found improvement in social relationships, that is through social contacts, related to accessible furniture and fixtures (Est. .09) as well as sufficient lighting (Est. .11). Also, other insufficient self-perceived indoor barriers, like toilet space in the bathroom (OR 46.7), height of the toilet (OR 25.0), shower space (OR 29.0), and the accessibility of the tub or shower (OR 8.0), increased the risk for low community participation as investigated by Yang & Sanford (2011) (CI not reported).

Tomsone et al. (2013) found a positive association between objectively measured barriers in the entrance and self-perceived health among those who were ADL independent in a Latvian sample (Est .009 (CI .03 to .15)), while barriers indoors were negatively associated with self-perceived health among those who were ADL independent in a Swedish sample (Est −.08 (CI -.14 to −.02)). No associations were found between self-reported health and accessibility of the indoor housing environment (Tsuchiya-Ito et al., 2019).

### Non-interventions targeting entrances

Four studies investigated the impact of entrance features on aspects of health such as body functions, quality of life, P-ADL, and I-ADL. The studies focused either on the walking surface leading to the building (Clarke, 2014) or walk-up buildings, that is family houses with more than one floor without an elevator (García-Esquinas et al., 2017; Pérez-Hernández et al., 2018; Tomioka et al., 2018).

Three studies focused on body functioning and had either a longitudinal, (Pérez-Hernández et al., 2018) or a cross-sectional study design (Clarke, 2014; García-Esquinas et al., 2017). Two studies used the Short Physical Performance Battery (SPPB) (García-Esquinas et al., 2017; Pérez-Hernández et al., 2018), and both concluded that there was no association between decreased body functioning and older adults living in a walk-up building. However, Clarke (2014) concluded that stairs at the entrance increase the risk of difficulties going outside independently (OR 1.52; CI 1.21 to 1.91).

Finally, three studies focused only on I-ADL by either using the Lawton & Brody Scale (García-Esquinas et al., 2017; Pérez-Hernández et al., 2018) or the Tokyo Metropolitan Institute of Gerontology Index of Competence (TMIG-IC) (Tomioka et al., 2018). One study found that older women living in a walk-up building were less likely to have an I-ADL decline in comparison to men where no such effect was found (OR .72; CI .52 to .99) (Tomioka et al., 2018). However, none of the studies that used the Lawton & Brody Scale reported any association between I-ADL decline and living in a walk-up building (García-Esquinas et al., 2017; Pérez-Hernández et al., 2018).

## Discussion

This systematic review synthesized recent evidence (published 2010 or later) on the relationships between physical housing characteristics, housing accessibility, and several aspects of health among community-dwelling people aged 60 years and older. The studies (*N* = 15) were grouped into (1) interventions by home modification, (2) non-interventions targeting indoor features, and (3) non-interventions targeting entrances. Significant associations between physical housing characteristics and health outcomes were reported in most of the studies, but the overall quality of evidence was graded as low for all health outcomes.

A striking finding was the high variability in results and study designs. This variability can be illustrated by the main health outcome under study (including the two RCTs), that is ADL (*n* = 7). The three intervention studies all had ADL as an outcome. Only the pilot trial by Szanton et al. (2011) showed a statistically significant association between home modifications and fewer ADL difficulties. Even though Taylor et al. (2020) saw a positive tendency to effect home modifications through an improvement in the ability to perform ADL, the effect was not significant. Similarly, Mitoku & Shimanouchi (2014) reported that older adults who got their homes modified decreased less in their ADL ability at each follow-up compared to those who did not get home modifications, but the differences were not significant. The longitudinal non-intervention study targeting indoor features found significant associations between housing accessibility problems and dependence in ADL (Slaug et al., 2017). Furthermore, one of the studies targeting entrances reported that women who lived in a walk-up building were less likely to have a decline in ADL, while such an effect was not found among men. (Tomioka et al., 2018). However, the authors acknowledge that those results should be taken cautiously due to risk of bias in sampling and disproportionate exclusion of many participants with ADL decline from the analysis at follow-up. Two other studies targeting entrances reported no association between ADL decline and living in a walk-up building (García-Esquinas et al., 2017; Pérez-Hernández et al., 2018). These inconsistent results could also be reflective of different measures to capture both issues in the housing environment and the performance of ADL. Yet, even if the results differ between the studies, the findings can be interpreted as a tendency of support for an association between ADL decline and living in an inaccessible housing environment, as reported in earlier research (see e.g., Iwarsson et al., 2007). However, the evidence is not conclusive. There is thus a need for future studies using similar measures, to further investigate the impact that the housing environment may have on ADL performance among various groups of older people.

Regarding the variability in study designs, we found only two intervention studies and of the remaining thirteen only four were longitudinal. Therefore, the evidence for making a causal link between the built environment and health outcomes is weak. The variability in study designs may also reflect the prerequisite of representing the complexity of the needs of older people and the change in their housing environment over time (Garin et al., 2014). The scarcity of high-quality intervention studies could be based on the fact that they create challenges within environmental research (Resnik, 2008). First, ethical issues can arise through higher risks for the participants such as violating their privacy in their home environment. Second, the costs related to the implementation of RCTs and longitudinal studies in comparison to cross-sectional studies are high. Third, the lack of blinding of participants may create the risk that their responses are affected, and, therefore, increase the risk of intentional or unintentional bias. Thus, to capture the complexity of the housing environment of older adults and to provide more evidence of causality, well-designed RCTs and longitudinal studies are needed. That is, by the use of an active control trial, where the control group receives an established intervention in their home environment, while the experimental group receives a new intervention. As a methodologically strong alternative to RCTs, simulation models could be beneficial (Schmidt et al., 2022), since they have the potential to estimate impacts of interventions prior to their implementation and minimize the risks for participants.

With regard to the outcomes, it was surprising that none of the included studies focused explicitly on well-being among older adults. It needs to be noted however, that OECD (2020) considers life satisfaction to be part of the construct well-being and two of the included studies targeted life satisfaction (Kim et al., 2021; Tsuchiya-Ito et al, 2019). One reason for the paucity of studies could be, that well-being is complex and difficult to measure (Costa-Font, 2013) even though it has been considered important in earlier studies (Guo et al., 2021). Future studies should therefore be specific in how they define and operationalize the concept of well-being to increase clarity on how the measure has been used.

### Limitations

This systematic review has some weaknesses that should be mentioned. First, the heterogeneity of population-based studies targeting the physical environment challenged the application of the GRADE approach. This issue has already been recognized by the GRADE working group (Boon et al., 2021) with the result that they plan to develop new GRADE guidelines to address the current shortcomings. However, we strengthened our decisions regarding the quality of evidence of the included studies through joint discussions between all co-authors. Second, the complexity of interventions and the heterogeneity of health outcomes and sample locations targeting the physical housing environment made it difficult to distinguish the findings in the synthesis. That is, some of the studies used measures that covered an overarching domain of our interest, while certain items within that domain were outside of our scope, for example, housing quality. This issue was solved by consensus discussions where the authors decided to include or exclude specific items. Third, as we conducted our preliminary search for the PROSPERO application, we focused on older adults aged 65 years and older. As a result of the preliminary search we altered our inclusion criterion to 60 years and older, since we found that many studies used this criterion for older people, as suggested by the WHO (see WHO, 2015b). However, as we altered this criterion prior to the systematic literature search, no study inclusion was affected by the change.

### Implications and Conclusion

Even though significant associations between physical housing characteristics and health outcomes were reported in several studies, the overall quality of evidence was low. Consequently, there is a need for further studies with a stronger research design and higher methodological quality. In particular, randomized controlled trials and longitudinal studies covering the physical housing environment and well-being were identified as a substantial research gap that needs to be addressed in future studies to strengthen the body of evidence and to give adequate recommendations for decision-makers. Furthermore, the results suggest that housing accessibility issues are not mainly a national issue but are recognized in many countries. Therefore, it is suggested that stakeholders should focus more on the planning of accessible housing and aim for policy solutions on regional, national and international levels in order to support populations aging in place.

## Supplemental Material

Supplemental Material - The Relationship Between Physical Housing Characteristics, Housing Accessibility and Different Aspects of Health Among Community-Dwelling Older People: A Systematic Review Click here for additional data file. Supplemental Material for The Relationship Between Physical Housing Characteristics, Housing Accessibility and Different Aspects of Health Among Community-Dwelling Older People: A Systematic Review by Christina Heller, Maria Haak, Steven M. Schmidt, Carlos Chiatti, Lisa Ekstam, Maria H. Nilsson, and Björn Slaug in Journal of Aging and Health

## Notes

1 Please note: the p-value was not reported.

2 Please note: incorrect confidence interval, seems to be duplicated for another variable.
