# Supplementary material for: The Relationship Between Physical Housing Characteristics, Housing Accessibility and Different Aspects of Health Among Community-Dwelling Older People: A Systematic Review
Source: J Aging Health. 2023 May 18;36(1-2):120–32. doi: 10.1177/08982643231175367 (PMC10693737; doi:10.1177/08982643231175367)
Supplement: Supplemental Material - The Relationship Between Physical Housing Characteristics, Housing Accessibility and Different Aspects of Health Among Community-Dwelling Older People: A Systematic Review [file sj-pdf-1-jah-10.1177_08982643231175367.pdf]

APPENDICES

Appendix 1. Search terms according the PICO scheme.

Domain	Search terms	Search string exemplified	Exclusion criteria
**Population**	Older people (aged 65 and older)	Old*OR senior* OR adult OR frail* OR aged OR ageing OR aging OR geriatric* OR "old age"	Aged 60 years and younger
**Intervention**	Physical housing characteristics, housing accessibility	"Built environment*" OR housing OR "capacity of Building" OR "housing access*"	If the intervention is not related to any assessment of the housing environment
**Comparator**	Non-intervention		
**Outcome**	Aspects of health such as: **Body functions**	"Body function*"	
	**Perceived health/well-being, quality of life, life satisfaction**	"self-perceived health", "perceived health", **"**well-being[MeSH Terms]"**,** "wellbeing", "well being", "quality of life"[MeSH Terms] OR ("quality"[All Fields] AND "life"[All Fields]) OR "quality of life"[All Fields], "health related quality of life"	
	**Activities of daily living**	"activities of daily living"[MeSH Terms] OR ("activities"[All Fields] AND "daily"[All Fields] AND "living"[All Fields]) OR "activities of daily living"[All Fields] OR ("activity"[All Fields] AND "daily"[All Fields] AND "living"[All Fields]) OR "activity of daily living"[All Fields]; "ADL*"	
	**Social participation**	"social participation"[MeSH Terms] OR ("social"[All Fields] AND "participation"[All Fields]) OR "social participation"[All Fields]; "involvement", "social engagement", "social contribution"	
**Setting**	ageing in place, community dwelling, independent living	"Community dwelling", "ageing in place", independent living, "independent living facility" OR "supportive housing" OR "service housing" OR "old age home" OR "old age homes" OR "age-restricted community" OR "independent living" OR co-housing OR "retirement home" OR "retirement housing" OR "senior apartment" OR "living facilities" OR "Design For Environment" OR "Environment Design" OR "Architectural Accessibility"	inpatient settings such as nursing homes, hospitals, and assisted living
**Type of studies**	RCT´s, meta analysis, quasi-experimental, longitudinal-studies, literature reviews		conference proceedings, qualitative studies, policy papers, clinical trials focusing on drug therapy, animal studies, case reports
**Time limitation**		Filter function: 2010 – now	
**Filter**		NOT Covid-19 OR "assisted living" OR nutrition OR (MH "Palliative Care") OR "oral health"<br>Filter function: only studies in English<br>Filter function: Publication type: Academic journals<br>Filter function: age: adult: 19-44 years, middle aged: 45-64 years, aged, 80 & over, aged: 65+ years, all adult	Studies in other languages than English

Appendix 2. Relationships between housing characteristics and aspects of health.

Abbreviations: **CASP-19**=Control, Autonomy, Self-Realization and Pleasure scale, **CASPAR**= Comprehensive Assessment and Solution Process for Aging Residents, **CSPS**= Continuously scored lower extremity Summary Performance Score, **DAD**=the Disability Assessment for Dementia, **EQ-5D**= EuroQol-5 Dimension, **ICECAP-O**= ICEpop CAPability measure for Older people, **I-ADL**=Instrumental Activities of Daily Living, **MBR**= Maximal Balance Range Tests, **MoCA**= Montreal Cognitive Assessment, **P-ADL**=Personal Activities of Daily Living, **PARTS/M**=Participation survey/mobility, **PPA**= Physiological Profile Assessment, **Qol**=Quality of Life, **SPPB**= Short Physical Performance Battery, **S-IQCODE**=Spanish Informant Questionnaire on Cognitive Decline in the Elderly, **TMIG-IC**=Tokyo Metropolitan Institute of Gerontology Index of Competence
Note: significant values are bolded

Body functions

Nr	Authors	Study design	Follow-up	Effect/comparison	Exposure (assessment tool)	Outcome	Analysis	Effect size/measure of relationship
1	Clarke, 2014	Cross-sectional	Not applicable	Effect of bad housing features	Walking surface leading to building, ramp; stairs at entrance (study specific checklist)	Difficulty going out (study specific)	Logistic regression	**Stairs: OR 1.52; CI 1.21; 1.91**<br>Ramp: OR 1.27; CI 0.98; 1.66<br>(Several interaction effects also calculated)
2	García-Esquinas et al., 2017	Cross-sectional	Not applicable	Effect of bad housing features	Walk-up building, no elevator (single item)	(1) SPPB<br>(2) Mobility lim.<br>(3) Agility lim.	Linear regression<br>Logistic regression<br>Logistic regression	(1) Est -0.02; CI -0.22; 1.18<br>(2) OR 0.90; CI 0.72; 1.13<br>(3) OR 0.97; CI 0.78; 1.21
3	González et al., 2020	Cross-sectional	Not applicable	Effect of good housing features	Physical home environment, i.e., decoration and location of home (HOME)	(1) S-IQCODE<br>(2) MoCa<br>(cognitive function)	Logistic regression	**(1) OR 0.95; CI 0.92; 0.96**<br>(2) Not significant
8	Pérez-Hernández et al., 2018	Longitudinal	2 years	Effect of bad housing features	Walk-up building, no elevator (single item)	SPPB	Linear regression	Est -0.06; CI -0.26; 0.14
11	Taylor et al., 2020	Randomized control trial	1 year	Home modification intervention vs. control (care are as usual)	Home hazards, e.g., lack of railings, raised thresholds, lack of lighting (Home Safety Assessment Tool)	(1) PPA<br>(2) SPPB<br>(3) CSPS<br>(4) MBR	Linear regression	Between group difference<br>(1) Est 0.1; CI -0.4; 0.5<br>(2) Est 0.2; CI -0.4; 0.8<br>(3) Est -0.06; CI -0.8; 0.2<br>(4) Est -8; CI -20; 4
15	Yang & Sanford, 2011	Cross-sectional	Not applicable	Effect of bad housing features	Physical home environment, barriers and facilitators (CASPAR)	Mobility limitation, ease-difficulty (study specific)	Spearman correlation	**Corr; Up/down stairs 0.303**<br>**Corr; Home space 0.364**<br>**Corr; Pathways 0.276**<br>**Corr; Door 0.297**<br>**Corr; Toilet 0.268**<br>**Corr; Tub/shower 0.257**<br>**Corr; Kitchen space 0.391**<br>**Corr; Kitchen appl 0.443**<br>**Corr; Kitchen upper cabin 0.269**<br>**Corr: Kitchen lower cabin 0.627**<br>**Corr; Bedroom space 0.468**<br>**Corr; Bedroom bed 0.393**<br>**Corr; Bedroom closet 0.570**

Self-perceived health

Nr	Authors	Study design	Follow-up	Effect/comparison	Exposure (assessment tool)	Outcome	Analysis	Effect size/measure of relationship
13	Tomsone et al., 2013	Cross-sectional	Not applicable	Effect of bad housing features, i.e., in Latvia and Sweden (respectively)	Physical barriers in the home environment, e.g., high thresholds, narrow door openings, stairs without handrails (Housing Enabler)	SF-36 single item (stratified by country and level of ADL dependence)	Ordinal regression	Entrances Latvia<br>**ADL Indep.; Est 0.09 CI 0.03; 0.15**<br>ADL Dep.: Est -0.07; -0.18; 0.04<br>Entrances Sweden<br>ADL Indep.: Est 0.03; CI -0.03; 0.09<br>ADL Dep.: Est -0.05; CI -0.05; 0.004<br>Indoor Sweden<br>**ADL Indep.: Est -0.08; CI -0.14; -0.02**<br>ADL Dep.: Est -0.04; CI -0.09; 0.007<br>(All other not included in this analysis step)
14	Tsuchiya-Ito et al., 2019	Cross-sectional	Not applicable	Effect of bad housing features	Accessibility of housing environment, e.g., difficulty entering or leaving the home, unable to climb stairs, difficulty maneuvering within rooms, no railings although needed (Healthy housing environment)	Self reported health (Stratified by level of ADL dependence)	Logistic regression	ADL Indep.: OR 1.40; CI 0.91; 2.14<br>ADL Dep.: OR 1.46; CI 0.85; 2.48

Quality of life

Nr	Authors	Study design	Follow-up	Effect/comparison	Exposure (assessment tool)	Outcome	Analysis	Effect size/measure of relationship
3	González et al., 2020	Cross-sectional	Not applicable	Effect of good housing features	Physical home environment, i.e., decoration and location of home (HOME)	ICECAP-O	Logistic regression	**OR 6.54; CI 1.75; 24.46**
5	Leung et al., 2018	Cross-sectional	Not applicable	Effect of good housing features	Indoor built environment, space and distance; building services, e.g., lighting, ventilation; supporting facilities, e.g., handrails, color; barrier-free design (study specific checklist)	Overall QoL (study specific)	Unspecified regression analysis	**Est. Furniture and fixtures 0.092**<br>**Est. Lighting 0.149**<br>**Est. Handrails -0.125**<br>Est. Barrier-free design not reported (CI not reported)*
7	Nakhodaeezadeh et al., 2017	Cross-sectional	Not applicable	Effect of good housing features	Characteristics of entrance, hall, lounge, kitchen, double bedroom, single bedroom, alternative bathroom, cupboard, general items, and assistive technology (EVOLVE)	CASP-19	Spearman correlation	**Corr coeff 0.279**
10	Szanton et al., 2011	Randomized controlled trial (pilot)	24 weeks	Home modification intervention vs control group (attention sessions)	Physical barriers in the home environment, e.g., holes in floors, uneven carpeting, and lack of railings or banister (Client Clinician Assessment protocol)	(1) EuroQOL<br>(2) EQ-5D	Difference in means between groups	**(1) Cohen D 0.89**<br>**(2) Cohen D 0.48**<br>(CI not reported)
11	Taylor et al., 2020	Randomized controlled trial	1 year	Home modification intervention vs. control (care are as usual)	Home hazards, e.g., lack of railings, raised thresholds, lack of lighting (Home Safety Assessment Tool)	EQ-5D	Linear regression	Est. 0.02; CI -0.04; 0.07

Life satisfaction

Nr	Authors	Study design	Follow-up	Effect/comparison	Exposure (assessment tool)	Outcome	Analysis	Effect size/measure of relationship
4	Kim et al., 2021	Cross-sectional	Not applicable	Effect of good housing features	Perceived housing accessibility and usability (study specific checklist)	To what degree participants were satisfied with their life	Linear regression	**Est. 0.22; CI -0.015; 0.04***
14	Tsuchiya-Ito et al., 2019	Cross-sectional	Not applicable	Effect of bad housing features	Accessibility of housing environment, e.g., difficulty entering or leaving the home, unable to climb stairs, difficulty maneuvering within rooms, no railings although needed (Healthy housing environment checklist)	How would you rate your life satisfaction in the last 3 days? (stratified by level of ADL dependence)	Logistic regression	ADL Indep.: OR 1.08; CI 0.73; 1.60<br>ADL Dep.: OR 1.30; CI 0.77; 2.16

*Note: Significant finding reported in the original article, but inconsistent with CI which seems to be duplicated from another variable

Personal ADL (P-ADL) and Instrumental ADL (I-ADL)

Nr	Authors	Study design	Follow-up	Effect/comparison	Exposure (assessment tool)	Outcome	Analysis	Effect size/measure of relationship
2	García-Esquinas et al., 2017	Cross-sectional	Not applicable	Effect of bad housing features	Walk-up building, i.e. no elevator (single item)	Lawton & Brody Scale (I-ADL)	Logistic regression	OR 1.33; CI 0.87; 2.03
6	Mitoku & Shimanouchi, 2014	Longitudinal	1 year<br>2 years<br>3 years	Home modification vs no home modification	Housing adaptations, e.g., installation of handrails, elimination of floor height differences, change of lavatory basin, change of floor materials, change of door etc. (Study specific protocol)	Progression of frailty, including mortality (P-ADL)	Chi-square	After 1 year: 48.6% vs. 58.7%<br>After 2 years: 62.9% vs. 71.9%<br>After 3 years: 79.5% vs. 84.8%
8	Pérez-Hernández et al., 2018	Longitudinal	2 years	Effect of bad housing features	Walk-up building, i.e. no elevator (single item)	Lawton & Brody Scale (I-ADL)	Logistic regression	OR 1.26; CI 0.82; 1.92
9	Slaug et al., 2017	Longitudinal (with a simulation modeling design)	1 year	Effect of bad housing features, i.e., in Germany and Sweden (respectively)	Physical barriers in the home environment, e.g., high thresholds, narrow door openings, stairs without handrails; measure of housing accessibility problems (Housing Enabler)	(1) Cooking<br>(2) Shopping<br>(3) Cleaning<br>(4) Transport.<br>ADL staircase (I-ADL)	Logistic regression	Germany<br>**(1) OR 1.004; CI 1.000; 1.008**<br>**(2) OR 1.003; CI 1.000; 1.007**<br>(3) OR 1.001; CI 0.998; 1.005<br>**(4) OR 1.005; CI 1.001; 1.008**<br>Sweden<br>**(1) OR 1.005; CI 1.002; 1.009**<br>**(2) OR 1.004; CI 1.001; 1.008**<br>**(3) OR 1.011; CI 1.007; 1.015**<br>**(4) OR 1.004; CI 1.001; 1.007**
10	Szanton et al., 2011	Prospective randomized control pilot trial	24 weeks	Home modification intervention vs control group (attention sessions)	Physical barriers in the home environment, e.g., holes in floors, uneven carpeting, and lack of railings or banister (Client Clinician Assessment protocol)	(1) Lawton & Brody Scale (I-ADL)<br>(2) ADL staircase (P-ADL)	Comparison of ADLs with difficulties	**(1) P-ADL: Cohen's Delta 0.63**<br>**(2) I-ADL: Cohen's Delta 0.62**<br>(CI not reported)
11	Taylor et al., 2020	Randomized control trial	1 year	Home modification intervention vs. control (care are as usual)	Home hazards, e.g., lack of railings, raised thresholds, lack of lighting (Home Safety Assessment Tool)	DAD: Disability assessment for dementia (P-ADL)	Linear regression	Est. -3.3; CI -8.7; 2.2
12	Tomioka et al., 2018	Population-based longitudinal cohort study	3 years	Effect of bad housing features, i.e., in men and women (respectively)	Walk-up building, i.e. no elevator (single item)	TMIG-IC (I-ADL)	Logistic regression	Men: OR 0.90; CI 0.71; 1.14<br>**Women: OR 0.72; CI 0.52; 0.99**

Social participation

Nr	Authors	Study design	Follow-up	Effect/comparison	Exposure (assessment tool)	Outcome	Analysis	Effect size/measure of relationship
5	Leung et al., 2018	Cross-sectional	Not applicable	Effect of good housing features	Indoor built environment, space and distance; building services, e.g., lighting, ventilation; supporting facilities, e.g., handrails, color; barrier-free design (study specific checklist)	Social relationships (study specific)	Unspecified regression analysis	**Est. Furniture and fixtures 0.09**<br>**Est. Lighting 0.11**<br>Est. Handrails Not reported<br>Est. Barrier-free design Not reported<br>(CI not reported)
15	Yang & Sanford, 2011	Cross-sectional	Not applicable	Effect of bad housing features	Physical home environment, e.g., height and location of toilet (CASPAR)	PARTS/M (community participation)	Unspecified regression analysis	**OR bathroom, toilet space: 46.7**<br>**OR bathroom, toilet: 25.0**<br>**OR bathroom, tub/shower space: 29.0**<br>**OR bathroom, tub/shower: 8.0**<br>Other areas Not significant*
